# Acne keloidalis nuchae in Asian: A single institutional experience

**DOI:** 10.1371/journal.pone.0189790

**Published:** 2017-12-14

**Authors:** Kiyong Na, Sang Ho Oh, Sang Kyum Kim

**Affiliations:** 1 Department of Pathology, Severance Hospital, Yonsei University College of Medicine, Seoul, Republic of Korea; 2 Department of Dermatology, Severance Hospital, Yonsei University College of Medicine, Seoul, Republic of Korea; Kinki Daigaku, JAPAN

## Abstract

Acne keloidalis nuchae, a type of folliculitis involving the back of the neck, is common in black men, although rare cases have been reported in patients of other ethnicities. We analyzed the clinicopathological features of acne keloidalis nuchae in 17 Asians. Patients’ age at the time of presentation ranged from 20 to 69 years. Most patients experienced the disease over 2 years (range, 3 months–20 years); follow-up data were available for 11 (65%) patients (range, 2–95 months). Nine (53%) patients had comorbidities, but none had a history of other skin disease or a family history of acne keloidalis nuchae. Macroscopically, seven (41%) patients had multiple erythematous pustulopapular lesions, and 10 (59%) had a single large plaque. Histopathologically, deep scarring folliculitis containing naked hair shafts was identified. In all cases, inflammation was most severe in the upper two-thirds of the dermis, and the differences in pustulopapular and plaque lesions were more prominent in the peri-inflammation area. Of the seven patients with plaque lesions treated with steroids alone or steroids and cryotherapy, three experienced plaque reduction. Acne keloidalis nuchae occurring in Asian patients frequently present with typical clinicopathological features, and therefore in spite of very low incidence the diagnosis of this disease entity should be considered in idiopathic scarring folliculitis of the posterior neck.

## Introduction

Acne keloidalis nuchae (AKN), also known as folliculitis keloidalis nuchae [1], is a form of chronic scarring folliculitis that is most prevalent in young black men. Although it is generally accepted that the lesion is not a keloid and is not associated with acne vulgaris and that the lesion may occur beyond the nuchal area [2], these terms are still most commonly used. Early AKN lesions are characterized by pustules and hard, erythematous papules on the occipital region of the scalp and the posterior region of the neck. Later, AKN lesions may coalesce into keloid-like plaque with destruction of the hair follicles, causing hair loss. Abscess and purulent discharge may develop with secondary infections in advanced cases [3].

AKN in patients of other ethnicities is rare, and only a few cases have been reported [4, 5]. Recently, we treated AKN in several Korean men who had progressively scarring folliculitis in the posterior nuchal area. Because AKN in Asian patients has not been reported in the literature, we investigated the clinicopathological features of AKN in patients diagnosed at our institution. Our findings suggest that AKN can be considered in the diagnosis of progressively scarring skin lesions of the occipital or posterior nuchal regions in Asian patients, although the incidence is rare.

## Materials and methods

**Fig 1:**
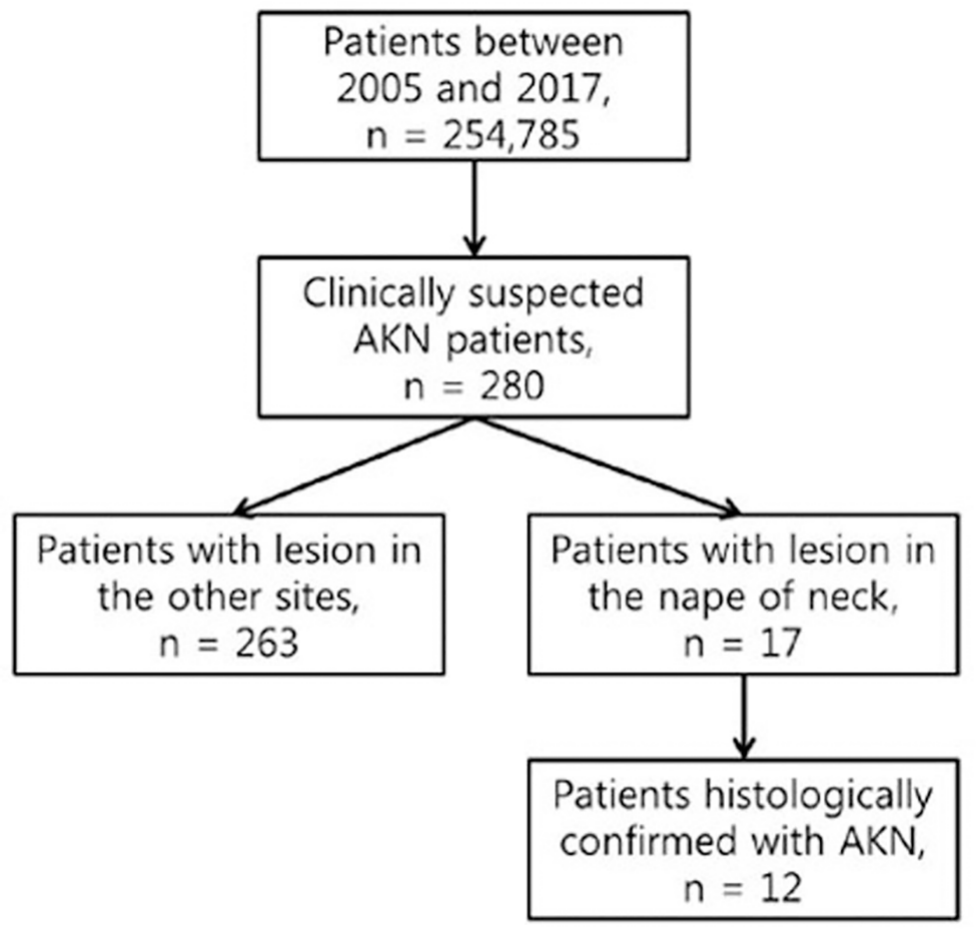
Patient selection diagram.

We retrospectively searched the database at Severance Hospital (Seoul, Republic of Korea) to identify patients with acne keloidalis nuchae diagnosed between 2005 and 2017 (Fig 1). Of the 280 patients identified, 17 had occipital or posterior neck lesions, and we reviewed their medical records for demographic, clinical, and histological information.

Histopathological samples, including 11 punch biopsy specimens and one excision specimen, were available for 12 (71%) out of 17 patients with acne keloidalis nuchae. The specimens were vertically sectioned to the epidermal surface, fixed in 10% neutral buffered formalin, and embedded in paraffin blocks. From each formalin-fixed, paraffin-embedded block, 4-μm sections were cut and stained with hematoxylin and eosin. All available specimen slides were examined by routine light microscopy.

All methods and experimental protocols using human tissue were carried out in accordance with relevant guidelines and regulations approved by the Institutional Review Board of Severance Hospital (4-2017-0547). The Institutional Review Board waived the written informed consent requirement because this retrospective study had a minimal risk to the patients (risk level I).

## Results

### Clinical features

Table 1 summarizes the clinical features of the 17 patients. All the patients were Korean men. Patients’ age at the time of presentation ranged from 20 to 69 years. Age distribution of disease onset was 17–40 years (13/17, 76%) or >50 years (4/17, 24%). Most patients had disease duration over 2 years (range, 3 months–20 years).

**Table 1:** Clinical features of 17 Korean men with acne keloidalis nuchae.

Case	Age	Duration	Site	Hair	Macroscopic finding	Disease history before presentation	Comorbidities	Follow-up
1	20	3 yr	Post. scalp	Kinky	Multiple erythematous papules	Pain and itching Transient improvement after ILTAI	None	NA
2	20	3 yr	Post. scalp		Multiple erythematous papules	Itching; Gradually progressing	None	Improved inflammation after oral steroid, minocycline, and ILTAI (12 mo)
3	20	2 yr	Post. scalp		Multiple erythematous papules	Itching; Gradually progressing	Gynecomastia	Improved inflammation after isotretinoin and ILTAI (6 mo)
4	21	1 yr	Post. scalp		Multiple erythematous papules and pustules	Itching; Gradually progressing	None	Improved inflammation by minocycline and ILTAI (8 mo)
5	26	2 yr	Post. neck	Kinky	Erythematous plaque	Repeated folliculitis; No improvement after laser therapy and ILTAI	None	Plaque reduction after cryotherapy and ILTAI (70 mo)
6	28	7 yr	Post. neck	Kinky	Multiple erythematous papules	Itching Alleviation and aggravation after ILTAI	Metabolic syndrome	Improved inflammation after cryotherapy and ILTAI (2 mo)
7	32	8 yr	Post. neck	Kinky	Erythematous plaque	Itching No improvement after ILTAI	None	Plaque reduction after ILTAI; patient requested excision (18 mo)
8	37	10 yr	Post. scalp		Multiple papules and plaque	Itching; Gradually progressing	Diabetes mellitus Ischemic heart disease	NA
9	45	10 yr	Post. neck		Erythematous plaque	No improvement after laser therapy and ILTAI	Renal cell carcinoma	NA
10	45	2 yr	Post. scalp		Multiple erythematous papules	Itching; Gradually progressing	Ischemic heart disease Metabolic syndrome	NA
11	52	4 yr	Post. scalp		Erythematous plaque	Gradually progressing	Diabetes mellitus	NA
12	52	13 yr	Post. scalp	Kinky	Erythematous plaque	Itching; No improvement after ILTAI	Metabolic syndrome	Minimal change after ILTAI (2 mo)
13	55	6 mo	Post. scalp		Erythematous plaque	Gradually progressing	None	Softening of the plaque after ILTAI Episode of staphylococcal abscess (36 mo)
14	58	20 yr	Post. scalp	Kinky	Erythematous plaque	Gradually progressing No improvement after laser therapy	HTN, Peptic ulcer	Minimal change after ILTAI (6 mo)
15	58	8 mo	Post. scalp		Erythematous plaque	Gradually progressing	None	Alleviation and aggravation after ILTAI (95 mo)
16	60	5 yr	Post. scalp		Multiple erythematous papules and plaque	No improvement after ILTAI	None	Alleviation and aggravation after ILTAI and isotretinoin Episode of staphylococcal abscess (29 mo)
17	69	3 mo	Post. Scalp	Kinky	Multiple erythematous papules	Itching; Transient improvement after topical steroid	Dyslipidemia	NA

Patients received various treatments before presentation, including topical steroid application, local steroid injection, or laser therapy. Overall, the lesions gradually progressed with repeated episodes of folliculitis, although some patients experienced transient alleviation of symptoms. Nine (53%) patients had comorbidities: metabolic syndrome (3), diabetes mellitus (2), renal cell carcinoma (1), hypertension (1), hyperlipidemia (1), and gynecomastia (1). None of the patients had a history of other skin disease or a family history of AKN.

**Fig 2:**
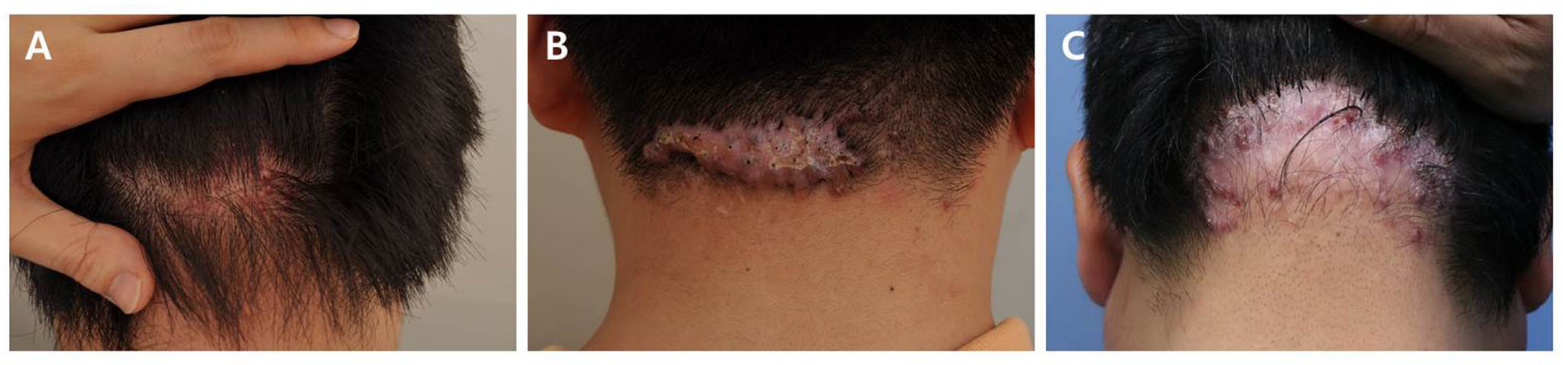
Clinical features of acne keloidalis nuchae (AKN) in 17 Korean patients. Follicular papules progressed to keloidal plaques with loss of hair. (a) 20-year-old man developed a lesion 3 months ago. (b) 26-year-old man developed a lesion 2 years ago. (c) 54-year-old man developed a lesion 13 years ago.

Thirteen (76%) patients had occipital lesions, and four (24%) patients had posterior nuchal lesions. Seven (41%) patients had multiple erythematous pustulopapular lesions (3–7 mm), and 10 (59%) patients had a single large plaque with crusts on the hair follicle openings (Fig 2). No hair growth was observed inside the thick plaque. Punch biopsies were performed in three patients with pustulopapular lesions and eight patients with a plaque lesion.

Four patients with pustulopapular lesions received intralesional triamcinolone injection (ILTAI) and either antibiotics or cryotherapy. They experienced improvement of the inflammation during the follow-up period.

Seven patients with a plaque lesion received ILTAI alone or combinations of ILTAI and cryotherapy. The plaque lesion softened and shrank in three of these patients. One of them underwent excision after the plaque lesion improved. The other four patients did not show improvement in plaque size or texture and experienced repeated episodes of plaque aggravation and alleviation. Two of them developed staphylococcal abscesses, which were treated by incision and drainage.

Follow-up data were available for 11 (65%) patients. The follow-up period ranged from 2 months to 95 months (median: 12 months; interquartile range: 30 months).

### Histological features

**Fig 3:**
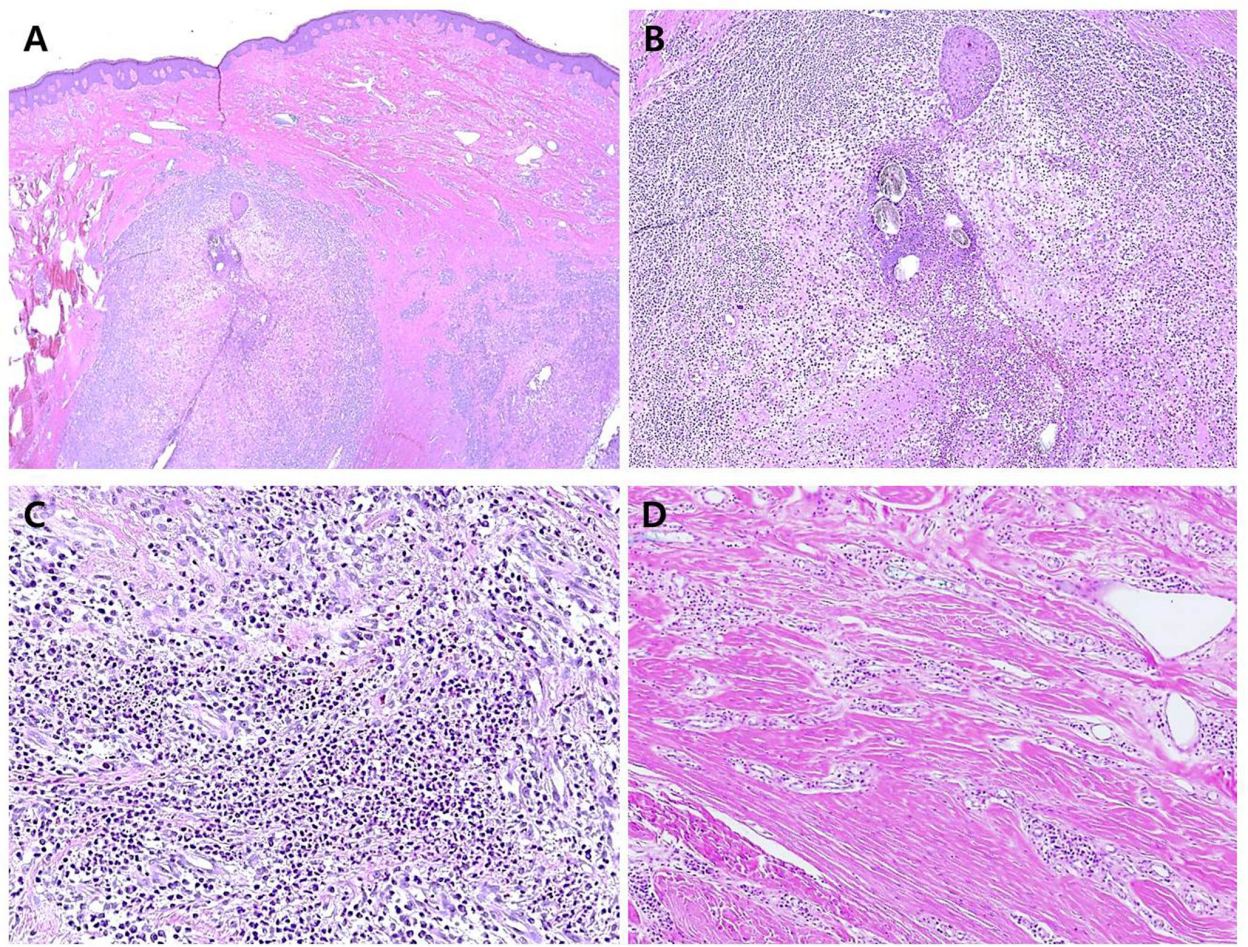
Histological features of acne keloidalis nuchae (AKN) in 17 Korean patients. Excised AKN specimen stained with hematoxylin and eosin. (a) Low-power view of specimen showing ruptured hair follicle in the mid-third portion of the dermis and irregularly arranged compact collagen bundles (magnification, 40×). (b) Some naked hair shafts and remaining follicular epithelium admixed with acute and chronic inflammatory cells (magnification, 100×). (c) Perifollicular region infiltrated by neutrophils, histiocytes, lymphocytes, eosinophils, and plasma cells (magnification, 200×). (d) Compact collagen bundles and dilated vasculature with lymphoplasmacytic infiltrate (magnification, 200×).

The epidermis exhibited hyperkeratosis, acanthosis, and bulbous rete ridges in all 12 specimens (Fig 3A). Aggregation of neutrophils, red blood cells, and bacterial colonies were occasionally observed in the corneal layer. In both pustulopapular and plaque lesions, there was a completely or partially ruptured hair follicle in the mid-third portion of the dermis. The partially ruptured hair follicle contained fragmented hair shafts, abundant keratins, basophilic debris, neutrophils, and bacterial colonies (Fig 3B). The remaining follicular epithelium showed focal neutrophilic infiltration with keratinocyte necrosis and leakage of keratinous materials into the dermis. The perifollicular region was infiltrated by histiocytes, lymphocytes, eosinophils, and plasma cells. In completely ruptured hair follicles, neutrophilic aggregation and infiltration of histiocytes, lymphocytes, and plasma cells replaced the follicular structure (Fig 3C). At the periphery of inflammation, islands of follicular epithelium, several multinucleated giant cells, and naked hair shafts were identified. The sebaceous gland was not observed in any specimen. The hair follicles in the deeper dermis were intact.

Dermal collagen was markedly thickened, and the extracellular matrix between collagen bundles was decreased (Fig 3D). In three pustulopapular lesion specimens, abundant fibroblastic proliferation in fibromyxoid stroma was observed at the periphery of the inflammatory foci. The remaining dermis showed dilated blood vessels and perivascular mild lymphohistiocytic infiltrate. The thickness of dermal collagen and the space of the extracellular matrix were preserved. In eight plaque lesion specimens, there was sparsely cellular and dense fibrosis at the periphery of the inflammation. The peri-inflammatory dermis showed dilated blood vessels and perivascular dense plasmacytic infiltrate.

Many hair follicles in the excision specimen showed different stages of inflammation. The predominant pattern was an infiltration of neutrophils, lymphocytes, histiocytes, and plasma cells in the ruptured hair follicle. However, some of hair follicles only showed focal neutrophilic infiltration in the epithelium of architecturally intact hair follicles. Other areas showed huge inflammatory collections consisting of plasma cells and some naked hair shafts.

## Discussion

The prevalence of AKN in black patients ranges from 0.7% to 13.6% [6]. During our study’s 12-year period, there were 17 patients with AKN out of 254,785 new patients in the dermatology department in our institution, indicating the rarity of AKN in Asian patients (0.007%). All of our patients were male, which is consistent with epidemiological features in black patients (male to female ratio, 20:1) [4]. One study showed that AKN is rare in black patients before puberty and after the age of 50 years [7]. Similarly, Adegbidi et al. demonstrated that about 90% of patients were younger than 40 years [6]. In our study, 76% of patients were younger than 50 years, and none of our patients experienced AKN before puberty. The age distribution of our patients was generally similar with that of black patients, although the group with late-onset AKN was slightly larger in our study. This discrepancy may be due to the small number of the patients in our series, and our findings are not conclusive.

Several theories have been suggested regarding the pathogenesis of AKN. Although most patients do not have a family history of AKN, the prevalence of AKN in black patients suggests that genetic susceptibility is a crucial factor. In addition, the prevalence of AKN in postpubertal men implies that androgen plays an important role in AKN pathogenesis [1, 7].

Chronic mechanical irritation to the nuchal and occipital area, such as frequent haircuts or friction from helmets or collars [8], also could trigger inflammation. Indeed, AKN is more prevalent in African-American football players than in other African Americans [9]. Three young patients in our series experienced the first symptoms of AKN during periods of military service. They were required to have frequent haircuts and to wear helmets, which might be related to the development of AKN.

Other etiologies, including antiepileptic agents, immunosuppressive agents, metabolic syndrome, and chronic infection, have been implicated in some patients [10–14]. Interestingly, about 30% of our patients had diabetes mellitus or metabolic syndrome.

Although pustulopapular lesions are considered to represent an earlier disease stage and plaque lesions are considered to represent a later disease stage, we did not find a relationship between disease duration and macroscopic appearance. Predisposing factors for plaque formation in AKN are still unknown, but it is generally accepted that patients with repeated folliculitis are at higher risk for plaque formation. Some studies have reported rapid progression of AKN plaque lesions after acid treatments [1]. One patient in our study experienced the rapid development of a plaque lesion after laser therapy. These observations indicate that physical or chemical stimulation may trigger the development of plaque lesions, although individual responses may differ.

Histopathologically, the AKN specimens in our study were characterized by folliculitis with destruction of the follicular epithelium, perifollicular granulomatous inflammation containing naked hair shafts, and perivascular inflammation. In all cases, inflammation was most severe in the upper two-thirds of the dermis, and the sebaceous glands were not observed. This finding is consistent with the results of microscopic examination of sequential transverse sections of AKN in a previous study by Herzberg et al. [15]. They demonstrated that the most intense inflammation occurred in the isthmus and lower infundibulum. Because this level of the hair follicle is at a merging point with the sebaceous gland [15], the absence of sebaceous glands at inflammatory foci suggests that the sebaceous gland is destroyed at an early stage of AKN. Destruction of the sebaceous gland in early-stage AKN supports the androgenic theory of AKN pathogenesis.

The biopsy specimens of pustulopapular and plaque lesions shared histopathological features with folliculitis and perifolliculitis. The excision specimen had many hair follicles showing different stages of inflammation, which included focal neutrophilic infiltration in intact hair follicles, admixture of various inflammatory cells in ruptured hair follicles, and dense plasmacytic aggregation containing islands of follicular epithelium. The neutrophilic infiltration of follicular epithelium probably occurs at an earlier stage in AKN, and plasmacytic aggregation probably occurs at a later stage. Coexistence of different stages of folliculitis in one lesion may explain why folliculitis in pustulopapular and plaque lesions can be similar. Differences in these lesions were most prominent in the peri-inflammatory area. Pustulopapular lesions exhibited intact collagen bundles and immature and cellular fibrosis in the peri-inflammatory area. In contrast, plaque lesions exhibited thickened collagen bundles, and mature and acellular fibrosis. In addition, pustulopapular lesions showed perivascular inflammation, predominantly with lymphocytes and histiocytes, whereas plaque lesions showed perivascular dense plasmacytic infiltration. In contrast to the histological features of folliculitis, the alterations in perivascular inflammatory cells and collagen architecture were correlated with the macroscopic appearance of the lesion.

In conclusion, we described the clinical and histological features of AKN in 17 Asian patients. Although AKN in Asians is rare, AKN should be considered in the diagnosis of chronic scarring folliculitis of the posterior neck or occipital region in Asian patients. Histopathological examination in our study revealed that different stages of inflammation may coexist in one lesion. The differences in pustulopapular and plaque lesions were more prominent in the peri-inflammatory area.
